# Development of Scales for Generative Concern and Generative Acts Among Older Singaporeans

**DOI:** 10.1093/geroni/igaa057.2954

**Published:** 2020-12-16

**Authors:** Rahul Malhotra, Ad Maulod, June May Ling Lee, Grand Hak-Land Cheng, Si Yinn Lu, Leng Leng Thang, Angelique W M Chan

**Affiliations:** 1 Duke-NUS Medical School, Singapore, Singapore; 2 The Open University of Hong Kong, Hong Kong, China; 3 Mailman School of Public Health, Columbia University, New York, New York, United States; 4 National University of Singapore, Singapore, Singapore

## Abstract

Generativity (concern in establishing and guiding the next generation) at older ages is increasingly relevant with population ageing and realization of older people as a resource for younger generations. Generativity encompasses two aspects, concern (attitudes/motivations for generativity) and acts (activities to enact/achieve generativity). Existing scales for generative concern and acts pertain to Western populations, limiting their valid measurement in Asian populations. We conducted 12 focus group discussions with 103 older adults in Singapore, to inform a conceptual model of generativity. A striking finding was the family-centric focus of generativity. It led to the development of content-validated scales for generative concern (38-items; e.g. I am concerned that younger people are too pampered) and generative acts (56-items; e.g. In past 3 months, how many times did you teach younger people right from wrong), in English, Mandarin, Malay and Tamil. Future work will establish their structural, convergent/divergent and predictive validity, and reliability.

